# Phenolic Compounds in *Ziziphus jujuba* Mill.: Advances in Distribution, Biosynthesis, and Pharmacological Activities

**DOI:** 10.3390/plants15081160

**Published:** 2026-04-09

**Authors:** Yuting Hu, Jiangtao Du, Yingying Fan, Fengjuan Liu, Weizhong He, Binbin Li, Xing Cui, Cheng Wang

**Affiliations:** 1 College of Smart Agriculture, Xinjiang University, Urumqi 830049, China; 107552303726@stu.xju.edu.cn; 2 Institute of Quality Standards & Testing Technology for Agri-Products, Academy of Agricultural Sciences of Xinjiang Uyghur Autonomous Region, Urumqi 830091, China; dujiangtao@xaas.ac.cn (J.D.); fyyxaas@sina.com (Y.F.); liufengjuan2050@126.com (F.L.); hewei198112@126.com (W.H.); libinbin@xaas.ac.cn (B.L.); cuixing96@163.com (X.C.); 3 Laboratory of Quality and Safety Risk Assessment for Agri-Products (Urumqi), Key Laboratory of Functional Nutrition and Health of Characteristic Agricultural Products in Desert Oasis Ecological Region (Co-Construction by Ministry and Province), Ministry of Agriculture and Rural Affairs, Urumqi 830091, China

**Keywords:** jujube, phenolic compounds, bioactive components, biosynthesis mechanism, pharmacological effects

## Abstract

Jujube (*Ziziphus jujuba* Mill.) is a functional food with both edible and medicinal properties. It is rich in various bioactive compounds and holds significant development value and application prospects in food nutrition, medicine, and health. This review systematically summarizes the research progress on the synthesis mechanism and pharmacological activities of phenolic compounds in jujube fruits, clarifies the composition of their main components, sorts out the research advances in extraction technologies of jujube phenolic compounds, and focuses on analyzing the content differences and distribution patterns across cultivars and tissue parts. On this basis, it examines the regulatory mechanisms of phenolic compound synthesis in depth, with a particular focus on elucidating the regulatory networks of genes and transcription factors involved in flavonoid biosynthesis. Meanwhile, this review comprehensively summarizes the pharmacological activities of phenolic compounds in jujube fruits, including antioxidant, anticancer, antibacterial, anti-inflammatory, and hypoglycemic effects. It also elucidates the molecular mechanisms underlying these bioactivities, such as regulating signaling pathways and scavenging free radicals. Finally, it analyzes the limitations of current research and proposes key directions for future development. This review provides theoretical support and a scientific basis for the in-depth development and utilization of jujube phenolic compounds as well as for the research and development of related functional foods and drugs.

## 1. Introduction

**Figure 1:**
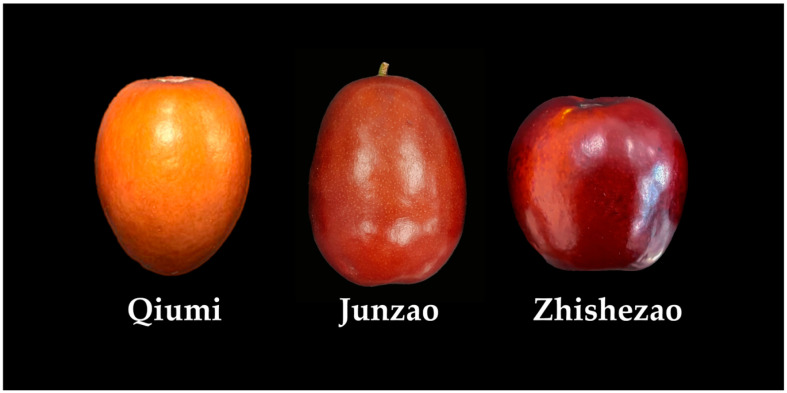
Photographs of jujubes (*Ziziphus jujuba* Mill.) at the ripening stage.

Jujube (*Ziziphus jujuba* Mill.) is a perennial deciduous tree belonging to the genus *Ziziphus* of the Rhamnaceae family, and occasionally presents a shrub form. This species is native to China and has been cultivated for more than 7000 years. China has abundant *Z. jujuba* germplasm resources, with more than 800 recorded cultivars, which are predominantly distributed in major producing regions such as Shaanxi, Shanxi, Xinjiang, Henan, Hebei, and Shandong provinces [1]. Its total annual fruit output exceeds 8 million tons, accounting for more than 98% of the global planting area and yield. As a representative plant with both edible and medicinal properties, jujube fruits are mainly consumed fresh or processed into dried products. Fresh jujubes are mainly used for direct eating to meet consumers’ daily dietary needs, while dried jujubes are widely utilized as a traditional Chinese medicinal herb due to their excellent storage resistance and medicinal efficacies including replenishing qi, nourishing blood and calming nerves, as well as invigorating the spleen and nourishing the stomach. Its medicinal application dates back more than 2000 years, and relevant records can be found in traditional Chinese medicine classics such as “Huangdi Neijing” and “Shennong Ben Cao Jing” [2]. In the current field of functional food development, jujube serves as the core raw material for deep-processed products including jujube wine [3], jujube tea [4], and jujube vinegar [5] owing to its high sugar content, rich flavor, as well as dual nutritional and medicinal values (Figure 1).

Jujube fruits are rich in nutrients, including carbohydrates, organic acids, vitamin C and various mineral elements [6,7,8,9]. Moreover, the fruits contain bioactive substances such as phenolic compounds, cyclic nucleotides (cAMP), alkaloids, saponins, and triterpenes [10,11,12,13]. With in-depth research on its functional components, jujube fruits exhibit a range of pharmacological effects. cAMP as a vital second messenger, participates in the regulation of various biological processes and exerts anti-allergic, liver-protective and cancer cell proliferation-inhibiting effects [14,15]. Alkaloids exhibit excellent antioxidant activity and anti-cancer effects [16]. Saponins can significantly inhibit the release of pro-inflammatory factors, thereby exerting anti-inflammatory activity. In addition, they also possess hypoglycemic activity, alleviate fatty liver lesions and show anti-tumor potential [17,18,19,20]. Triterpenoids also have functional activities such as anti-inflammatory and anticancer effects [21].

Phenolic compounds are key bioactive components in jujube fruits and are classified into two major categories, namely flavonoids and phenolic acids, which are accumulated in various tissue parts of jujube fruits. Pharmacological studies have asserted that phenolic compounds from jujube fruits have great application potential owing to their antioxidant, anticancer, anti-inflammatory, antibacterial and hypoglycemic properties, as well as their ability to alleviate heavy metal toxicity. To date, several reviews have systematically summarized the functional components and their pharmacological activities in jujube fruits. However, phenolic compounds have only been briefly mentioned as a subclass [22,23,24,25]. In addition, one review has provided a detailed summary of triterpenoids, including their dynamic changes in content, biosynthetic pathways, and pharmacological effects [26]. Nevertheless, a systematic review focusing specifically on phenolic compounds in jujube fruits is still lacking. Therefore, this review focuses on phenolic compounds in jujube fruits, systematically elaborating on their composition, dynamic changes in content, tissue distribution, extraction methods, synthetic regulation, and pharmacological mechanisms. It provides theoretical support for in-depth research on the nutritional and medicinal values of jujube phenolic compounds and serves as a reference for their industrial application in the food and pharmaceutical sectors.

## 2. Composition, Distribution and Extraction Processes of Phenolic Compounds

Jujubes are rich in diverse phenolic compounds, of which phenolic acids (including hydroxycinnamic and hydroxybenzoic acids) and flavonoids (such as flavones, flavonols, and flavanols) are the key bioactive phenolic components. These metabolites can undergo enzymatic interconversion via the phenylpropanoid pathway.

### 2.1. Analytical Methods for Phenolic Compounds

The Folin–Ciocalteu colorimetric method and the NaNO_2_-Al(NO_3_)_3_-NaOH spectrophotometric method are commonly employed for the determination of total phenolic content (TPC) and total flavonoid content (TFC). However, the high levels of reducing sugars and ascorbic acid present in jujube fruits possess strong reducibility, which can trigger non-specific chromogenic reactions with the Folin–Ciocalteu reagent, leading to the systematic overestimation of TPC values [27]. Similarly, compounds with an ortho-diphenol hydroxyl structure present in jujube fruits can react non-specifically with Al^3+^, resulting in systematic overestimation of TFC [28]. In contrast, high-performance liquid chromatography (HPLC) enables the accurate quantification of individual phenolic compounds, with superior specificity and precision. Accordingly, it has become the preferred approach for the qualitative and quantitative analysis of individual phenolic compounds [29].

### 2.2. The Content of Phenolic Compounds Is Influenced by Varietal Differences

TPC is strongly influenced by cultivar-specific variations [30]. Wang et al. examined 16 jujube cultivars from six major production regions in China, namely, Hebei, Xinjiang, Shanxi, Shaanxi, Shandong, and Henan provinces. The cultivars included ‘Ruoqiangzao’ (RQZ), ‘Jishanbanzao’ (JSBZ), and ‘Zhanhuangzao’ (ZHZ). The TPC was determined by the Folin–Ciocalteu colorimetric method, and the TFC was measured via NaNO_2_-Al(NO_3_)_3_-NaOH spectrophotometric method. Analyses revealed that the TPC in these cultivars ranged from 2.53 to 4.95 mg gallic acid equivalent (GAE)/g dry weight (DW), whereas the TFC varied from 1.25 to 4.25 mg rutin equivalent (RE)/g DW [31]. Zhang used 37 mature jujube cultivars from Shandong Province, including ‘Daguazao’ (DGZ), ‘Huizao’ (HZ), and ‘Tanzao’ (TZ), as experimental materials, and adopted the same method for the quantitative analysis of phenolic compounds. The results demonstrated that the TPC ranged from 8.45 to 16.33 mg GAE/g DW, and the TFC ranged from 6.00 to 41.18 mg RE/g DW [32]. Meanwhile, to effectively solve the problems of single cultivar composition and shortage of special-purpose cultivar resources in jujube cultivation, current research has conducted systematic determination on jujube germplasm resources from the main producing areas in Xinjiang, China. Zhang collected 76 varieties of jujube fruit in Kashgar, Xinjiang, and determined the content of phenolic compounds in jujube fruits using the exact same method as the aforementioned study. The results showed that the TPC of the fruits was 3.53–10.69 mg GAE/g fresh weight (FW), and the TFC was 1.25–12.50 mg RE/g FW [33]. In another study, the research team collected 142 jujube cultivars planted in the Tarim region of Xinjiang, and the measured TPC of jujube fruits ranged from 5.09 to 19.77 mg GAE/g FW, with the TFC ranging from 1.24 to 6.61 mg RE/g FW [34]. We also employed the Folin–Ciocalteu colorimetric method and NaNO_2_-Al(NO_3_)_3_-NaOH spectrophotometric method to analyze the phenolic compound contents in four jujube cultivars from Xinjiang namely ‘Suanzao’ (SZ), ‘HZ’, ‘Junzao’ (JZ) and ‘HamiDazao’ (HMDZ). The TPC ranged from 2.71 to 4.47 mg GAE/g DW, while the TFC varied between 1.29 and 2.78 mg RE/g DW.

To investigate the observed variations in TPC and TFC in jujube fruits reported in previous studies, we conducted a more in-depth analysis. It was found that, in addition to the influences of cultivar and geographical origin, differences in extraction and analytical methods may represent the primary causes underlying these variations. Specifically, on the one hand, differences in the polarity of extraction solvents exert significant effects on the extraction efficiency of phenolic compounds from jujube fruits. On the other hand, during the determination of TPC, variations in the ratio of Folin–Ciocalteu reagent to sodium carbonate, the dosage of the extract solution, and the detection wavelength can all alter the absorbance intensity of the chromogenic reaction, leading to systematic deviations [35]. Furthermore, differences in the dosage of chromogenic reagents, sodium hydroxide concentration, and constant-volume solvents used for TFC determination directly affect the complexation efficiency between flavonoids and Al^3+^. Overall, using 60% ethanol as the extraction solvent yields optimal results. For TPC determination, Folin–Ciocalteu reagent and 10% sodium carbonate solution are added at a volume ratio of 1:5, with detection carried out at 765 nm. For TFC determination, 5% sodium nitrate, 10% aluminum nitrate, and 4% sodium hydroxide are added sequentially, followed by volume adjustment to a constant volume with 60% ethanol and measurement at 510 nm. This combined protocol demonstrates satisfactory performance in both extraction efficiency and stability of the chromogenic system, and thus serves as a well-defined and reliable method for the analysis of TPC and TFC in jujube fruits.

### 2.3. The Content of Phenolic Compounds Is Influenced by the Tissue Site

Beyond the influence of cultivar, TPC distributions differ significantly among distinct jujube parts (peel, pulp, and seeds), with the peel accumulating the highest levels, followed by the pulp and seeds. Zhang et al. investigated the TPC in the peel, pulp, and seeds of three jujube cultivars, namely ‘Dongzao’ (DZ), ‘Muzao’ (MZ), and ‘HMDZ’, using the Folin–Ciocalteu colorimetric method. The results demonstrated that the TPC in all tissue parts of ‘DZ’ was significantly higher than those of ‘MZ’ and ‘HMDZ’. Specifically, the TPC in ‘DZ’ was 4.17 ± 0.24 mg GAE/g DW in seeds, 8.13 ± 0.72 mg GAE/g DW in pulp, and 32.80 ± 0.34 mg GAE/g DW in peel. In contrast, the corresponding TPC in ‘MZ’ was 2.89 ± 0.28 mg GAE/g DW (seeds), 5.93 ± 0.63 mg GAE/g DW (pulp), and 8.74 ± 0.19 mg GAE/g DW (peel), while those in ‘HMDZ’ were 2.28 ± 0.11 mg GAE/g DW (seeds), 5.57 ± 0.28 mg GAE/g DW (pulp), and 6.08 ± 0.50 mg GAE/g DW (peel). Notably, a consistent distribution pattern was observed across the three jujube cultivars, with the TPC in the peel being significantly higher than that in the pulp and seeds [36]. In another comparative study of six cultivars (‘JZ’, ‘DZ’, ‘Pingguozao’ (PGZ), ‘Fucuimi’ (FCM), ‘Jinlingyuanzao’ (JLYZ), and ‘Fengmiguan’ (FMG)), the TPC ranged from 18.11 to 21.45 mg GAE/g DW, and the TFC was 10.56–20.25 mg RE/g DW. Proanthocyanidins, (−)-epicatechin, (+)-catechin, and rutin were the dominant phenolic components, representing 58.60%, 16.08%, 13.56%, and 5.57% of the total phenolic compounds, respectively. Further studies revealed that the peel contained an average TPC of 32.30 mg GAE/g DW and an average TFC of 34.43 mg RE/g DW. In contrast, the pulp displayed substantially lower levels of both components. The average TPC in the pulp was 17.29 mg GAE/g DW, whereas the average TFC was 9.07 mg RE/g DW. The TPC in the peel was 1.87-fold higher than that in the pulp. For TFC, the peel showed more pronounced accumulation, which was 3.80-fold greater than that in the pulp. Furthermore, phenolic compounds are heterogeneously distributed within the pulp, with increased levels in the regions adjacent to the pit and pedicel [37]. Our experimental data further supported this perspective. Specifically, the TPC and TFC in different developmental stages (young, enlargement, white-ripe, semi-red, and full-red stages) and distinct parts (peel and pulp) of ‘Zhishezao’ (ZSZ) were determined. The findings indicated that the TPC in the peel ranged from 7.49 to 21.11 mg GAE/g FW, while that in the pulp varied from 4.69 to 6.99 mg GAE/g FW. For TFC, the content in the peel was in the range of 6.76–18.81 mg RE/g FW, whereas the corresponding value in the pulp was 0.71–2.41 mg RE/g FW. Collectively, these analyses revealed that both TPC and TFC in the peel were significantly higher than those in the pulp.

### 2.4. Phenolic Acids

**Figure 2:**
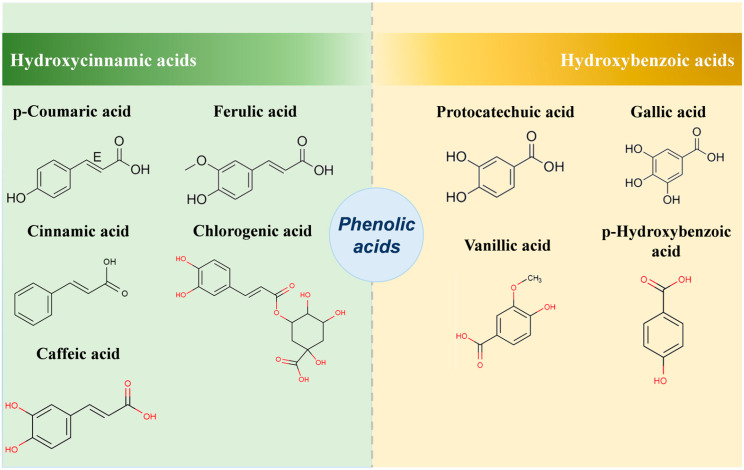
Main component structures of phenolic acids in jujube fruits.

Based on the analysis of the distribution and content of phenolic acids in different parts of the jujube fruit, they can be categorized into two subclasses: hydroxycinnamic acids and hydroxybenzoic acids. The former comprises p-coumaric acid, cinnamic acid, caffeic acid, chlorogenic acid, and ferulic acid, whereas the latter includes p-hydroxybenzoic acid, protocatechuic acid, gallic acid, and vanillic acid (Figure 2). Phenolic acids in the jujube fruit exist primarily in four forms: free, esterified, glycosidic, and insoluble-bound. Of these, the insoluble-bound phenolic acids are the predominant fraction, accounting for 40.9%, 48.3%, and 40.3% of the total phenolics in the peel, pulp, and seeds, respectively. Conversely, free phenolic acids constitute merely 6.1%, 7.5%, and 8.6% of total phenolics in the respective parts [38]. Xie et al. identified phenolic acids in the peel of seven jujube cultivars, namely, ‘Jixinzao’ (JXZ), ‘JZ’, ‘Mayazao’ (MYZ), ‘Yuancuizao’ (YCZ), ‘RQZ’, ‘Lingwuchangzao’ (LWCZ), and ‘Zhanhuadongzao’ (ZHDZ), at distinct developmental stages, using HPLC. Their findings established that the phenolic acid content was the highest at the white ripening stage in all cultivars, with ‘ZHDZ’ displaying the highest accumulation in the peel at this stage. Five phenolic acids were detected, which included gallic acid, chlorogenic acid, caffeic acid, p-coumaric acid and ferulic acid. Of these, caffeic acid (0.0368 ± 0.00108 to 0.231 ± 0.00572 mg/g DW), chlorogenic acid (0.0173 ± 0.00143 to 0.157 ± 0.00563 mg/g DW), and gallic acid (0.01089 ± 0.00068 to 0.137 ± 0.00637 mg/g DW) were the major components [39]. In another investigation, ultra-high performance liquid chromatography (UHPLC) was adopted to identify the phenolic acid composition of fully ripe fruits from 20 major jujube cultivars, including ‘JSBZ’, ‘Yongjihamazao’ (YJHMZ), ‘Yunchengxiangzao’ (YCXZ), ‘Guantanzao’ (GTZ), and ‘Yuanlingzao’ (YLZ). Gallic acid, chlorogenic acid, and ferulic acid were detected in all cultivars. Further analysis of phenolic acids in ‘HPZ’ at various developmental stages revealed that the phenolic acid content was the highest at the fruit expansion stage. Gallic acid (0.428 ± 0.00475 mg/g FW) and chlorogenic acid (0.0293 ± 0.00194 mg/g FW) were the predominant phenolic acids in ‘HPZ’ at this stage [40].

In summary, the phenolic acids in jujube fruits are mainly concentrated in the peel, existing primarily in the insoluble bound form, with gallic acid, chlorogenic acid, ferulic acid, and caffeic acid as the main components. However, regarding the significant differences in phenolic acid content observed in the aforementioned studies, in addition to considering differences in jujube varieties and the source of sample parts, differences in extraction and analytical methods may be the main factors leading to the differences in content. Specifically, on the one hand, the choice of extraction solvent affects the extraction efficiency of phenolic acids, 80% ethanol has limited ability to extract phenolic acids, while the methanol: water: formic acid (70:29:1, *v*/*v*/*v*) system can denature cell membranes due to its acidic nature, enhancing the interaction between the solvent and the compound, thereby improving the extraction rate of bound phenolic acids [41]. On the other hand, there are differences in the selection of chromatographic detectors and conditions among different studies. Compared with the traditional HPLC, UHPLC has higher separation sensitivity and resolution, which can more accurately quantify the phenolic acid components and further improve the reliability of the detection results of phenolic acid content. Therefore, comprehensively, the combination of acidified methanol for extraction and UHPLC for qualitative and quantitative analysis is currently a relatively optimal method for analyzing the phenolic acid components in jujube fruits.

### 2.5. Flavonoids

The backbone of flavonoids comprises 15 carbon atoms arranged in a C_6_-C_3_-C_6_ structure, where two aromatic rings (A and B) are linked by a C_3_ bridge. Most flavonoids are composed of three rings (A, B, and C) and can be classified into subclasses, including flavones, flavonols, flavanols, flavanones, flavanonols, anthocyanidins, and others, based on the cyclization, substitution, and oxidation patterns of the C_3_ unit.

A study identified and analyzed flavonoids in 37 jujube cultivars, which included ‘JXZ’, ‘HZ’, ‘JSXZ’, ‘Xiangzao’ (XZ), and ‘Qingxuyuanzao’ (QXYZ) using HPLC–diode array detector (HPLC–DAD). The findings signified that (+)-catechin and rutin were relatively abundant, and the highest contents were detected in ‘QXYZ’ at 0.653 mg/g DW and 0.039 mg/g DW, respectively [42]. In another research, HPLC-DAD was similarly employed to identify flavonoids in eight jujube cultivars (‘Dabailing’ (DBL), ‘JZ’, ‘Junyou’ (JY), ‘SZ’, ‘HZ’, ‘ZHZ’, ‘Fushuai’ (FS), and ‘FCM’) across five developmental stages (young, fruit core-hardening, green ripening, half-red maturity and complete red). Proanthocyanidins, catechin, and epicatechin were the major components, which were predominant at the young fruit stage [43]. Shi et al. utilized HPLC–DAD to identify 10 key flavonoids from the fruits of ‘Tailihong’ (TLH) and ‘JZ’, including five flavanols (procyanidin B1, procyanidin B2, procyanidin B3, (−)-epicatechin and (+)-catechin) and five flavonols (quercetin-3-galactoside, quercitrin-3-glucoside, quercetin-3-rutinose, quercetin-3-rhamnoside and quercetin) [44]. UHPLC–tandem mass spectrometry (UHPLC-MS/MS) was employed by Xue et al. to detect 122 flavonoids in ‘HPZ’ at the white period and red period, which encompassed 40 flavonols, 37 flavones, 12 anthocyanidins, 9 flavanones, 8 flavanols, 6 flavone carbonyl glycosides, 5 flavanonols, 3 isoflavones, and 2 chalcones. Further metabolic network analysis showed that high levels of cynaroside, (-)-epigallocatechin, (+)-gallocatechin, catechin and anthocyanins were accumulated at both stages [45]. In another study, Qiu et al. observed that flavanols were the most prevalent flavonoids in ‘DZ’ fruits and accounted for 88% of the total flavonoids, with (−)-catechin, epicatechin, and rutin being the major components [46].

Anthocyanins are crucial water-soluble pigments present in plants and primarily contribute to the red color observed in many fruits [47]. These pigments are widely accumulated in the peel of different jujube cultivars. A total of 15 anthocyanins were identified in the peel of ‘DZ’ using HPLC-DAD, of which delphinidin, malvidin 3-O-glucoside and delphinidin 3-O-glucoside demonstrated a significant hike in content during the ripening stage, implying that they are the key anthocyanins responsible for the red color of the jujube peel [48]. Li et al. determined the anthocyanidin content in the peel of ‘FMG’ and ‘TLH’ across all developmental stages and found that it ranged from 0.144 to 0.314 mg/g and from 0.137 to 0.345 mg/g, respectively. UHPLC-electrospray ionization-tandem mass spectrometry (UHPLC-ESI-MS/MS) was used to identify anthocyanidin compounds. A total of 12 anthocyanins were detected in the peel of ‘FMG’ and 13 in the peel of ‘TLH’. Specifically, cyanidin, cyanidin 3,5-O-diglucoside, pelargonidin, and peonidin played pertinent roles in the early coloring process of fruits, whereas apigeninidin chloride, cyanidin O-syringic acid, and delphinidin were involved in the complete coloring process [49].

**Figure 3:**
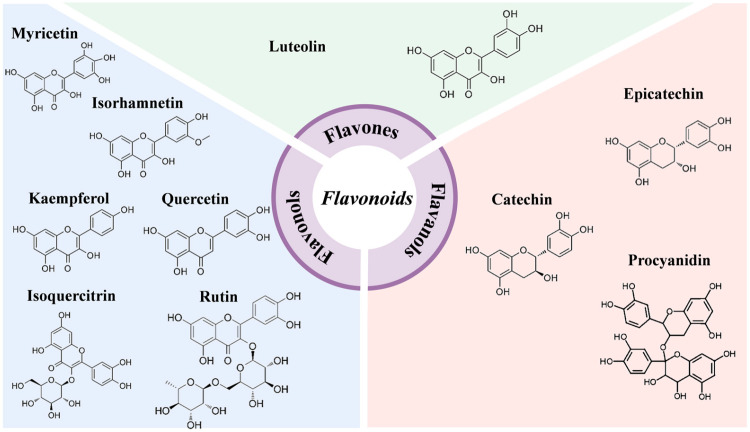
Main component structures of flavonoids in jujube fruits.

The above studies establish that (+)-catechin, epicatechin, anthocyanins and rutin are the predominant components in most jujube cultivars and that anthocyanins play a key role in jujube fruit coloring (Figure 3). However, regarding the significant differences in the types of flavonoids reported by different studies, in addition to the differences in varieties and developmental stages, systematic differences in analytical methods may be an important factor leading to this phenomenon. Specifically, on the one hand, the separation ability and sensitivity of detection techniques determine the identification effect of flavonoids. Traditional HPLC-DAD is a targeted analytical method, whose qualitative and quantitative analysis relies on standard substance comparison, and has the advantages of simplicity and economy in the accurate quantification of known main components. UHPLC-MS/MS has higher separation resolution, mass spectrometric qualitative sensitivity and non-targeted screening ability [50], and can comprehensively analyze the flavonoid metabolic profile of jujube fruits. On the other hand, different studies have differences in sample pretreatment and the selection of chromatographic conditions, which have a significant impact on the extraction efficiency and separation effect of different types of flavonoids, and further interfere with the identification results of flavonoids in jujube fruits.

### 2.6. Extraction Process

Organic solvent extraction, ultrasound-assisted extraction (UAE), microwave-assisted extraction (MAE), and deep eutectic solvent (DES)-assisted extraction are effective methods for isolating phenolic compounds from jujube. However, traditional organic solvent extraction usually requires multi-step operations using organic solvents (such as water, ethanol, and methanol) under high temperature and long-time conditions [51,52]. In addition, many organic solvents can lead to ionization, hydrolysis, oxidation, and degradation of flavonoid compounds. Some solvents are also hazardous to human health and raise significant environmental concerns [53].

One such innovation is the introduction of DESs, first proposed by Abbott et al. [54], which are regarded as a new class of green and sustainable solvents. DESs offer enhanced thermal stability, high extraction efficiency, and easy degradability, along with additional advantages including low toxicity, cost-effectiveness, and biodegradability [55]. At present, DESs have been successfully applied to the extraction of flavonoids from jujube fruits. Combining DES with ultrasound, microwave, or enzyme-assisted techniques can further improve extraction efficiency while reduce solvent consumption and energy consumption. Previous studies have investigated the effects of different extraction methods on the flavonoid compounds in ‘JZ’. The results indicated that the TFC obtained varied significantly among the methods: hot water extraction yielded only 3.61 mg/g, ethanol extraction resulted in 5.14 mg/g, UAE-DES extraction achieved 5.79 mg/g, and enzymatic hydrolysis-assisted DES extraction reached 6.85 mg/g. Among these strategies, the MAE-DES method exhibited the optimal performance, with the highest TFC of 8.03 mg/g. A comparison of the five extraction strategies demonstrated that DES-assisted extraction is an efficient approach for recovering TFC from jujube fruits. Furthermore, the study further examined the impact of the five methods on individual flavonoid monomers. The findings revealed that UAE-DES extraction effectively mitigated the degradation of flavonoid monomers during the isolation process, making it a more suitable and effective method for the extraction of flavonoid monomers from jujube [56].

Additionally, several green extraction techniques, including supercritical fluid extraction, bio-based solvent extraction, and ionic liquids combined with microwave, ultrasound or pressure-assisted techniques have been applied to the efficient separation of phenolic compounds in food. These efficient and green extraction technologies are expected to be applied to the extraction of phenolic compounds from jujube fruits in the future, thereby providing new ideas and technical support for their efficient separation and purification [57,58].

## 3. Synthesis Mechanisms of Phenolic Compounds

Elucidating the biosynthetic mechanisms of phenolic compounds in jujube fruits provides theoretical support for jujube variety improvement and further promotes their pharmacological applications. To date, research on the biosynthesis of phenolic compounds in jujube fruit has mainly focused primarily on flavonoids; studies on biosynthetic pathways and key regulatory genes of phenolic acids remain scarce. Flavonoids are synthesized from phenylalanine via the phenylpropanoid pathway, with the formation of chalcone being the first committed step [59].

**Figure 4:**
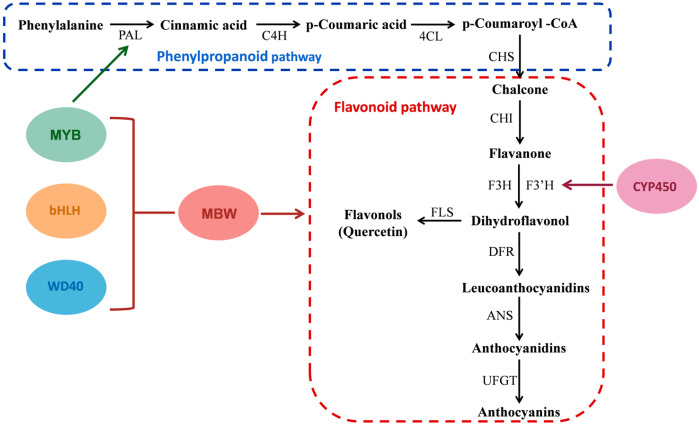
Metabolic pathway of flavonoids in jujube fruits (PAL: phenylalanine ammonia-lyase; C4H: cinnamate 4-hydroxylase; 4CL: 4-coumarate: coenzyme A ligase; CHS: chalcone synthase; CHI: chalcone isomerase; F3H: flavanone 3-hydroxylase; F3′H: flavonoid 3′-hydroxylase; FLS: flavonol synthase; DFR: dihydroflavonol 4-reductase; ANS: anthocyanidin synthase; UFGT: UDP-glucose: flavonoid 3-O-glucosyltransferase).

Phenylalanine is converted to cinnamic acid by phenylalanine ammonia-lyase (PAL), followed by hydroxylation to form p-coumaric acid under the action of cinnamate 4-hydroxylase (C4H). Subsequently, p-coumaric acid is transformed by 4-coumarate: coenzyme A ligase (4CL) to produce p-coumaroyl-CoA [60]. p-Coumaroyl-CoA is converted to chalcone by chalcone synthase (CHS), which is then transformed to flavanone by chalcone isomerase (CHI). Flavanone is further converted to dihydroflavonol via the action of flavanone 3-hydroxylase (F3H) and flavonoid 3′-hydroxylase (F3′H). Cytochrome P450 (CYP450) is one of the largest enzyme metabolic families occurring in plants and encodes F3H and F3′H, playing a crucial role in flavonoid biosynthesis. Dihydroflavonol is a key precursor for the synthesis of flavonols and anthocyanins. Flavonol synthase (FLS), a member of the 2-oxoglutarate-dependent dioxygenase family, is the critical enzyme required for the biosynthesis of flavonols and other flavonoids. This enzyme converts dihydroflavonol to flavonol by regulating gene expression, enzyme activity, and substrate specificity. Dihydroflavonol is transformed to leucoanthocyanidins by dihydroflavonol 4-reductase (DFR), followed by conversion to anthocyanidins via the action of anthocyanidin synthase (ANS). Finally, anthocyanidins form anthocyanins under the catalysis of UDP-glucose: flavonoid 3-O-glucosyltransferase (UFGT) (Figure 4).

Integrated metabolomic and transcriptomic analyses of flavonoid compounds during jujube fruit development were conducted in a study, identifying nine potential genes associated with their biosynthesis. Of these, only the expression trend of *ZjCYP98A9a-89* was aligned with the alterations in flavonoid content in the fruit, and its transient overexpression significantly enhanced flavonoid accumulation, confirming the involvement of *ZjCYP* in flavonoid biosynthesis [61]. Xue et al. cloned an FLS homologous gene (*ZjFLS*) from ‘HPZ’. Quantitative reverse transcription-polymerase chain reaction (qRT-PCR) revealed that *ZjFLS* had the highest expression level in white-ripe-stage fruits. Heterologous expression of *ZjFLS* in *Arabidopsis thaliana* Col-0 resulted in a TFC of 2.510 mg/g FW in transgenic lines, which was 3.67 -fold that of the wild type, asserting the ability of the *ZjFLS* gene to promote flavonoid biosynthesis [62]. Furthermore, an investigation showed that FLS can catalyze the conversion of dihydroflavonol to quercetin. Transcriptomic and metabolomic analyses of samples from ‘JZ’ across all growth stages led to the identification of two genes (*ZjFLS1* and *ZjFLS2*) with high correlation coefficients with the quercetin content. Furthermore, qRT–PCR and HPLC analyses were performed, which revealed that the relative expression levels of *ZjFLS1* and *ZjFLS2* decreased continuously during fruit development, aligning with the pattern of quercetin accumulation. When *ZjFLS1* and *ZjFLS2* were transiently overexpressed, the content of quercetin-3-O-rutinoside was significantly increased; in contrast, when they were silenced, the synthesis of quercetin-3-O-glucoside and quercetin-3-O-rutinoside was inhibited. These findings also confirm that *ZjFLS* is a key gene in flavonoid biosynthesis in jujube fruits [63].

Anthocyanins are the major pigments in the peel of jujube fruits. Zhang et al. obtained ‘DZ’ peels at three different developmental stages (white, semi-red, and full-red) and conducted transcriptomic analysis. The findings indicated the gradual activation of UFGT genes involved in anthocyanin synthesis and accumulation (*LOC107427037*, *LOC107406597*, and *LOC107427038*) during fruit maturation. As ripening progressed, anthocyanidins were converted by UFGT genes to key anthocyanins in the peel, including delphinidin, malvidin 3-O-glucoside, and delphinidin 3-O-glucoside [48]. DFR is an NADPH-dependent enzyme, and its expression exhibited a significant positive correlation with anthocyanin content (*p* < 0.01), suggesting that DFR is a key gene regulating anthocyanin biosynthesis [47].

**Figure 5:**
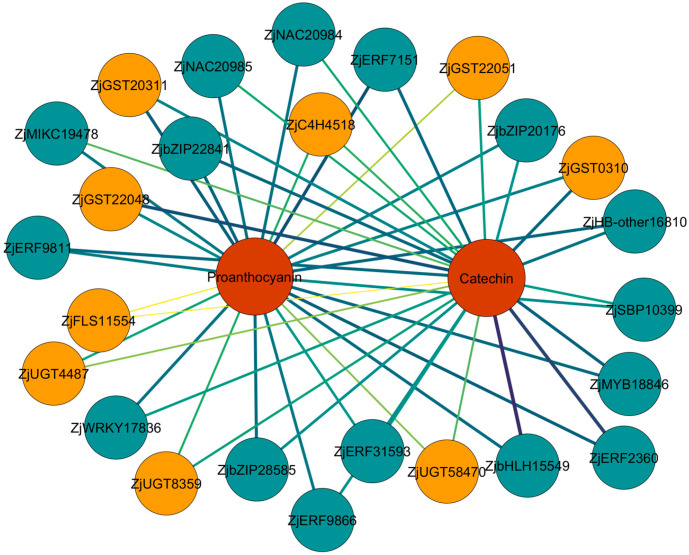
Gene co-expression network diagram of transcription factors, structural genes, and metabolites. Metabolites are marked with red circles, structural genes with orange circles, and transcription factors with green circles. Edge colors indicate correlation, with darker colors signifying higher correlation coefficients. The list of genetic information is provided in Table 1.

The transcription factors MYB, bHLH, and WD40 are intricately linked to flavonoid biosynthesis. Ji et al. identified 93 MYB genes in jujube fruits at five developmental stages (young fruit, enlargement, white-ripe, semi-red, and full-red stages), of which 17 were negatively correlated and 39 were positively correlated with TFC [64]. WD40 lacks a DNA-binding domain and does not exhibit transcriptional activity, routinely interacting with MYB and bHLH to form the MYB-bHLH-WD40 (MBW) complex. This complex regulates the expression of CHI, F3H, F3′H, DFR, ANS, and UFGT, thereby promoting anthocyanin biosynthesis [65]. Another study on the mechanism of anthocyanin biosynthesis in the fruit peel identified two structural genes (*ZjANS* and *ZjUGT79B1*) and eight transcription factors (ZjMYB113, ZjMYB5, ZjGL3a, ZjGL3b, ZjTT8, ZjWDR1, ZjWDR2, and ZjWDR3) associated with anthocyanin synthesis. Of these, ZjMYB5, ZjTT8, and ZjWDR3 activated the transcriptional activity of the promoters of *ZjANS* and *ZjUGT79B1*, thus increasing the synthesis of cyanidin-3-O-rutinoside and peonidin-3,5-O-diglucoside [66]. These findings establish that the MBW complex plays a pivotal role in anthocyanin biosynthesis. In the regulatory network of flavonoid biosynthesis, in addition to the MBW transcriptional complex, other transcription factor families, such as ZjERF, ZjSBP, ZjMIKC, ZjNAC, ZjWRKY, ZjbZIP, and ZjHB, can regulate the expression of flavonoid biosynthesis-related genes, thereby affecting their synthesis and accumulation [67] (Table 1) (Figure 5).

## 4. Pharmacological Activities and Mechanisms of Action of Phenolic Compounds

Jujube has been extensively employed in traditional Chinese medicine, where it exerts disease-preventive and adjuvant therapeutic effects by modulating metabolic processes and immune functions [68]. Of the various bioactive components in jujube, phenolic compounds extracted from fruits have been reported to possess diverse pharmacological activities.

### 4.1. Antioxidant Activity

To date, numerous studies have demonstrated that phenolic compounds in jujube fruits are closely associated with their antioxidant capacity. Zhao et al. used 95% ethanol as the extraction solvent to isolate bioactive compounds from seven major jujube cultivars at maturity, including ‘Goutouzao’ (GTZ), ‘Banzao’ (BZ), ‘Pozao’ (PZ), and ‘JZ’. The antioxidant activities of these extracts were systematically evaluated using in vitro experimental design (based on phosphomolybdenum radical scavenging activity, superoxide radical scavenging activity, and hydroxyl radical scavenging activity) and ex vivo experimental design (including antihemolytic activity and the inhibition of lipid peroxidation in rat liver homogenates). The results showed that phenolic compounds were the main bioactive constituents in jujube extracts, with their content ranging from 4.54 to 12.99 mg GAE/g DW. Moreover, the extracts of all seven jujube cultivars exhibited significant antioxidant activities in the above-mentioned assays. To further clarify the correlation between phenolic compounds and antioxidant activity, a correlation analysis was performed in the study. The results revealed that phenolic content was significantly positively correlated with antioxidant capacity (*p* < 0.05) [51]. Li et al. conducted crude extraction on the air-dried fruits of ‘JSXZ’ using 95% ethanol. After liquid–liquid phase separation, petroleum ether (PE) extract, ethyl acetate (EA) extract and water-saturated n-butanol (n-BuOH) fraction were obtained separately. Subsequently, the antioxidant activities of the three extracts were evaluated via three assays: reducing power, DPPH radical scavenging, and β-carotene bleaching inhibition assay. The results demonstrated that the ranking of antioxidant activity among different JSXZ extracts was fully consistent with that of their TPC and TFC. Specifically, the EA extract exhibited the optimal antioxidant potential, with TPC of 86.50 ± 0.87 mg GAE/g DW and TFC of 289.28 ± 3.39 mg RE/g DW; followed by the n-BuOH fraction, with TPC of 21.59 ± 0.12 mg GAE/g DW and TFC of 113.69 ± 0.79 mg RE/g DW; the PE extract showed the weakest antioxidant capacity, with TPC of 8.12 ± 0.37 mg GAE/g DW and TFC of 84.73 ± 0.82 mg RE/g DW [69]. To further explore the variation in antioxidant capacity during different fruit developmental stages of jujube, Wang et al. employed the Ferric Reducing Antioxidant Power (FRAP) assay to conduct an in-depth analysis of the antioxidant activity in ‘JSBZ’ fruits at the white mature, half-red, and full-red stages. Their results not only verified the significantly positive correlation between TPC, TFC and antioxidant activity (*p* < 0.05), but also clarified that the TPC, TFC and FRAP antioxidant activity of ‘JSBZ’ all peaked at the white mature stage, and showed a downward trend with the increase in fruit maturity [70]. In addition, another study used fruits from seven jujube cultivars, including ‘JXZ’ and ‘JZ’, and systematically analyzed their antioxidant capacity by dividing them into three developmental stages (green, yellow or reddish, and red) based on peel color. The results of the study also confirmed that the antioxidant activity of jujube was significantly positively correlated with the TPC and TFC, and further verified that the antioxidant capacity was the highest at the white mature stage. Meanwhile, the study also found that the phenolic compound content and antioxidant capacity in the peel were significantly higher than those in the pulp. Moreover, among different cultivars, the phenolic compound contents of ‘JZ’, ‘LWCZ’, and ‘ZHDZ’ were significantly higher than those of other cultivars [39].

### 4.2. Anticancer Activity

Reactive oxygen species (ROS) serve as crucial signaling molecules for evaluating cancer cell viability. Li et al. investigated the anticancer effects of phenolic extracts from ‘JZ’ by treating hepatocellular carcinoma (HepG2) cells with varying concentrations of the extracts. The findings indicated that the phenolic compounds effectively inhibited HepG2 cell proliferation and promoted apoptosis by inducing ROS generation and disrupting cellular homeostasis. Furthermore, a negative correlation was observed between the concentration of phenolic compounds and the survival rate of HepG2 cells. Of the various components, TPC, quercetin-3-rutinoside, and procyanidin B1 were determined to be the predominant anticancer constituents, with respective contents of 2.027 mg GAE/g DW, 0.253 mg/g DW, and 0.0486 mg/g DW [71]. In yet another research, the inhibitory activities of three different extracts (aqueous, EA, and hydroalcoholic extracts) from jujube fruits against leukemia cells were assessed. The observations demonstrated that the EA extract significantly reduced the viability of human acute myelogenous leukemia cells (KG-1) and human pre-B acute lymphoblastic leukemia cells (NALM-6) in a concentration-dependent manner, thus exerting a potential therapeutic effect on acute leukemia. Phenolic compounds and flavonoids were identified to be the chief bioactive components in this extract, with TPC of 151.64 ± 0.03 mg GAE/g and TFC of 1.21 ± 0.02 mg RE/g, respectively [72]. Shan et al. isolated free phenolic extracts (FPEs) and bound phenolic extracts (BPEs) from jujube pulp using acetone extraction and alkali digestion methods, and evaluated their anti-hepatoma activities via in vitro (HepG2 hepatoma cell viability assay) and in vivo (nude mouse tumor model) experiments, respectively. The results demonstrated that only BPEs could induce excessive ROS accumulation in HepG2 cells, thereby exerting anti-hepatoma effects, and no significant toxicity was observed during long-term administration in nude mice. Further component analysis revealed that p-coumaric acid and ferulic acid were the main active constituents of BPEs, which might enhance the anti-hepatoma efficacy through synergistic effects. These findings indicated that BPEs are promising to be developed as potential therapeutic agents for hepatoma [73].

### 4.3. Anti-Inflammatory Activity

As a prototypical endotoxin, lipopolysaccharide (LPS) stimulates macrophages and triggers an inflammatory response. A study investigating the anti-inflammatory activity of phenolic compounds from the peel and seeds of ‘GTZ’ revealed that peel-derived phenolics exhibited better antioxidant and anti-inflammatory abilities than those from seeds. Specifically, the TPC and TFC in the peel were observed to be 119.969 ± 0.811 mg GAE/g and 94.108 ± 0.771 mg RE/g, respectively. Quercetin, galangin, and rutin were identified as the major phenolic compounds in the peel, with respective contents of 11.7 mg/g, 4.61 mg/g, and 3.15 mg/g. The phenolic compounds in the peel significantly reduced the production of nitric oxide (NO). These compounds alleviated LPS-induced inflammatory responses by inhibiting the activation of mitogen-activated protein kinase (MAPK) and downregulating the protein levels of inducible NO synthase and cyclooxygenase-2, thus suppressing the translocation of LPS-induced nuclear factor-κB (NF-κB) [74]. Furthermore, in a subsequent study, polyphenols from ‘HZ’ peel were employed to treat LPS-stimulated RAW264.7 macrophages. The results demonstrated that peel polyphenols inhibited the activation of MAPK and NF-κB pathways, and significantly reduced the production of NO, tumor necrosis factor-α (TNF-α), interleukin-6 (IL-6), and interleukin-1β (IL-1β). In addition, they alleviated cellular oxidative stress and inflammation by activating the nuclear factor erythroid 2-related factor 2 (Nrf2) signaling pathway. The activities of antioxidant enzymes, including superoxide dismutase (SOD), catalase, and glutathione peroxidase (GSH-Px) were enhanced, which in turn decreased the production of ROS and malondialdehyde (MDA), and inhibited the phosphorylation of extracellular signal-regulated kinase (ERK), c-Jun N-terminal kinase (JNK), and p38. P-coumaric acid, (+)-catechin, and rutin were the major phenolic components in the HZ peel, with respective contents of 95.17 ± 4.62 mg/g, 318.16 ± 12.46 mg/g, and 261.57 ± 8.72 mg/g [75]. Wei et al. reported that phenolic extracts from ‘JZ’ could alleviate intestinal inflammation by significantly downregulating the expression of pro-inflammatory cytokines, and markedly increase mucin expression, thereby exerting a positive regulatory effect on the repair of intestinal barrier damage. In addition, these extracts effectively maintained the homeostasis of the gut microbiota and ultimately significantly ameliorated the clinical symptoms of mice with ulcerative colitis, providing a novel potential strategy for the prevention of ulcerative colitis [76].

Liver injury is one of the most common inflammatory responses. Persistent liver injury can lead to liver diseases, functional decline, liver fibrosis, and cirrhosis, and even induce HepG2 in severe cases. Acetaminophen (APAP) administration induces acute liver injury in mice. Huang et al. showed that flavonoid compounds extracted from ‘JSXZ’ augmented the activities of SOD and GSH-Px and inhibited the production of MDA in the livers of APAP-injured mice, thereby effectively alleviating APAP-induced oxidative liver injury. Moreover, these flavonoids decreased downregulated the expression levels of NO, TNF-α, IL-6, and IL-1β, and activated the Nrf2 signaling pathway, ultimately exerting a protective effect against liver injury in mice [77]. Chronic alcohol exposure inhibit hepatic fatty acid oxidation and promote adipogenesis in hepatocytes, thereby leading to severe alcoholic liver injury. Liu et al. evaluated the hepatoprotective effects of the water extract of ‘JSXZ’ (WEJ). The results indicated that WEJ is rich in phenolic compounds. Specifically, WEJ effectively reduced the activities of hepatocyte injury marker enzymes in serum, alleviated alcohol-induced fatty liver disease under long-term alcohol consumption, and oral administration of WEJ exerted significant protective and therapeutic effects against alcohol-induced liver injury in mice. Furthermore, WEJ significantly reduced the MDA content in liver tissues and suppressed the production of IL-6 and TNF-α in the liver, thereby ameliorating alcohol-induced inflammatory injury in hepatic tissues [78].

### 4.4. Antibacterial Activity

Phenolic compounds derived from jujube exhibit antibacterial activity against both Gram-positive and Gram-negative bacteria. In a study, the disc diffusion method was used to assess the antibacterial activity of phenolic extracts from ‘JZ’ against three pathogenic microorganisms, namely *Staphylococcus aureus* (*S. aureus*), *Escherichia coli* (*E. coli*) and *Candida albicans* (*C. albicans*). The results suggested that these phenolic extracts effectively inhibited the proliferation of all three pathogens. TPC, quercetin-3-rutinose, and procyanidin B1 were the primary antibacterial components and were quantified to be 2.027 mg GAE/g DW, 0.253 mg/g DW, and 0.0486 mg/g DW, respectively. In addition, the antibacterial efficacy was found to be positively associated with the concentration of phenolic extracts [71]. Another study extracted phenolic compounds from jujube fruits using methanol as the extraction solvent. The antibacterial activity of jujube extracts against *Salmonella Typhimurium (S. Typhimurium)* and *E. coli* O157:H7 was evaluated via the microbroth dilution method. The results showed that the minimum inhibitory concentrations (MICs) of jujube extracts against *E. coli* O157:H7 and *S. Typhimurium* were 625 µg/mL and 1250  µg/mL, respectively, and the extracts exhibited effective antibacterial activity at their respective MICs [79]. Wang et al. purified flavonoid monomers from the crude extract of jujube fruits and identified five flavonoids, namely, epicatechin, quercetin, rutin, isoquercitrin, and hyperin. Antibacterial activity assays revealed that epicatechin, quercetin, and rutin exhibited significant inhibitory effects against *E. coli, S. aureus, Shigella,* and *Pseudomonas aeruginosa (P. aeruginosa)*. Of the various flavonoids, quercetin displayed the most potent inhibitory activity against *E. coli*, *Shigella*, and *P. aeruginosa*, whereas rutin demonstrated the strongest inhibitory effect on *S. aureus*. Isoquercitrin was observed to inhibit the growth of *S. aureus* and *Bacillus subtilis (B. subtilis)*, whereas hyperin displayed antibacterial activity only against *B. subtilis*. Further studies indicated that the antibacterial activity of these flavonoids was pronounced at pH < 6 and decreased gradually with increasing pH values. Complete loss of antibacterial activity was noted when the pH ranged from 8 to 10. The presence of metal ions, such as Na^+^, Ca^2+^, K^+^, Fe^2+^, and Mg^2+^, enhanced the antibacterial efficacy of these flavonoid compounds [80].

*Alternaria alternata* is a plant saprophytic and pathogenic fungus that induces diseases such as black rot in jujube fruits, thereby impairing food safety. The phenylpropanoid pathway is the primary metabolic route for the biosynthesis of phenolic compounds. Yang et al. demonstrated that treatment of ‘DZ’ with L-methionine (Met) significantly enhanced its antibacterial and antifungal activities, leading to improved resistance against black rot disease. Mechanistically, Met modulates the phenylpropanoid pathway by upregulating the expression of differentially expressed genes, including PAL, CYP73A, 4CL, and CAD, which in turn promotes the synthesis of phenolic compounds. Specifically, TPC exhibited a 1.58-fold increase, whereas TFC displayed a 1.06-fold increase. Concurrently, the accumulation of key differentially expressed metabolites, namely, trans-ferulic acid, salicylic acid, delphinium pigments, and hesperidin-7-neohesperidin, was significantly upregulated [81].

### 4.5. Other Pharmacological Effects

Phenolic compounds from jujube demonstrate a wide range of additional pharmacological activities. Insufficient insulin secretion results in elevated blood glucose levels and the development of type 2 diabetes mellitus (T2DM). Liao et al. explored the antidiabetic effect of jujube polyphenols via oral administration to T2DM model mice. The researchers found that these polyphenols significantly reduced fasting blood glucose, glycated hemoglobin, insulin, and homeostatic model assessment of insulin resistance (HOMA-IR) levels in diabetic mice, while concurrently alleviating the symptoms of polyphagia associated with T2DM [82]. Another study explored the effects of jujube on obesity and blood glucose levels in mice fed with a high-fat and high-sugar diet. Studies have shown that intake of fruits rich in phenolic compounds can inhibit adipocyte differentiation and proliferation, and effectively suppress the utilization efficiency of ingested energy, thereby exerting anti-obesity effects. Meanwhile, it can reduce serum lipid-related indicators and ameliorate dyslipidemia. In addition, jujube also significantly decreases fasting blood glucose and insulin levels in mice, thus effectively regulating blood glucose homeostasis [83].

Phenolic extracts derived from jujube peel have been shown to effectively alleviate aluminum- and cadmium (Cd)-induced toxicity. Chen et al. extracted phenolic compounds from ‘DZ’ peels and found that they could effectively ameliorate aluminum-induced oxidative stress, restore the activities of antioxidant enzymes in aluminum-poisoned rats, and exert neuroprotective effects to mitigate aluminum-induced damage to the nervous system. In addition, the extracts significantly attenuated the reduction in hematological parameters caused by aluminum toxicity, improved hematological abnormalities, and thereby effectively alleviated the overall systemic toxic injury induced by aluminum [84]. In terms of alleviating Cd-induced toxicity, Li et al. extracted BPEs from the peels of ‘JZ’ and compared the effects between the Cd exposure group and the BPEs improvement groups in *Caenorhabditis elegans (C. elegans)*. Their findings demonstrated that BPEs from jujube significantly ameliorated Cd-induced behavioral deficits in *C. elegans* and effectively alleviated Cd-triggered intestinal damage as well as lipid and lipofuscin accumulation. Mechanistically, BPEs reduced lipid accumulation in both *C. elegans* and *E. coli* OP50 by regulating the expression of genes linked to fatty acid synthesis [85].

## 5. Conclusions and Perspectives

This review summarizes the composition, content variation, extraction technologies, biosynthesis mechanism, and pharmacological activities of phenolic compounds in jujube fruits, aiming to provide a reference for the effective development and application of jujube phenolic compounds in nutraceutical, healthcare and pharmaceutical fields.

TPC and TFC are influenced by cultivar differences and tissue parts (peel, pulp and seed), with the highest accumulation in the peel. Gallic acid, chlorogenic acid, ferulic acid, caffeic acid, (+)-catechin, epicatechin, anthocyanins and rutin are the major phenolic compounds in jujube fruits. Pharmacological studies have demonstrated that jujube phenolic compounds exhibit antioxidant, anticancer, anti-inflammatory, antibacterial and other biological activities, making them an important source of natural medicines. Elucidating the accumulation patterns and biosynthesis mechanisms of jujube phenolics provides a theoretical basis for breeding jujube cultivars with high phenolic content and creating novel germplasms resources, and further facilitates the development and application of their pharmacological activities. Accumulating studies have confirmed that the phenylpropanoid pathway is the main metabolic pathway for flavonoid biosynthesis in jujube fruits. Dihydroflavonol, the key precursor for the biosynthesis of flavonols and anthocyanins, can be catalyzed by FLS to generate flavonols, and by DFR to produce anthocyanins. In addition, interactions among transcription factors form the MBW complex, which regulates the expression of key genes involved in the flavonoid biosynthesis pathway.

However, current research on phenolic compounds in the jujube fruit have mainly focused on commercial cultivars. Studies on the functional properties of local specialty varieties remain relatively scarce, making it difficult to fully reflect the diversity of phenolic resources within *Ziziphus* plants. In addition, phenolic acids and flavonoids are two core branch metabolites of the phenylpropanoid pathway. The potential synergistic or competitive regulatory network between their biosynthesis pathways remains unclear, and the regulatory mechanism underlying phenolic acid biosynthesis in jujube fruits has not yet been elucidated. These key research gaps severely restrict the progress of jujube cultivar improvement. Furthermore, studies on the pharmacological properties of phenolic compounds in jujube remain insufficient. Currently, most relevant studies have focused on a few common pharmacological functions. The diverse biological effects and mechanisms documented in other medicinal plants have not been systematically examined in the jujube fruit, which greatly limits the in-depth development and utilization of its medicinal value.

Therefore, future studies should expand the scope of study materials to include jujube samples from diverse producing regions, developmental stages, and growth environments to clarify the composition and accumulation patterns of phenolic compounds across varieties and ecological conditions. With respect to extraction technologies, multiple efficient and green extraction techniques have been widely used for the preparation of phenolic compounds in foods, and are expected to be applied for the targeted extraction and efficient utilization of phenolic compounds from jujube in the future. In addition, it is critical to systematically elucidate the main biosynthetic pathways of phenolic acids, validate the functions of their key regulatory genes, and analyze the mutual regulatory mechanisms between phenolic acid and flavonoid synthesis pathways. These measures will help improve our understanding of the molecular network governing phenolic compound synthesis in the jujube fruit. Moreover, comprehensive studies on the medicinal mechanisms, modes of action, and potential synergistic effects of phenolic compounds in the jujube fruit are required to clarify their potential applications in disease prevention and treatment, thereby laying a theoretical foundation for the development of novel and environmentally friendly functional therapies.

The implementation of the aforementioned research will provide systematic theoretical support for targeted variety improvement in jujube, thereby augmenting the nutritional and medicinal value of its fruits. Simultaneously, it will provide raw material assurance and technical support for the functional development of jujube-related products, facilitating the expansion of the jujube industry from conventional fresh consumption to functional foods, pharmaceutical excipients, and other high-value sectors. Ultimately, these advances will effectively enhance the overall competitiveness of the jujube industry.

## 6. Literature Search Methods

This review systematically retrieved relevant research literature on phenolic compounds in jujube fruits published from November 2001 to 2026. The retrieval platforms covered mainstream Chinese and international academic databases and search engines, including PubMed, CNKI, ScienceDirect, and Web of Science. To ensure the comprehensiveness of literature collection, the retrieval was conducted in both Chinese and English, with the core keywords set as: jujube, phenolic compounds, flavonoids, phenolic acids, extraction methods, biological activities, pharmacological effects, biosynthesis mechanism, and cultivars. The types of finally included literature covered original research articles, review articles, and academic dissertations, among which there were 10 review articles, 70 original research articles, and 5 master’s dissertations.

## Figures and Tables

**Table 1:** Gene annotation in gene co-expression network diagram [67].

Gene ID	Enzymes or Transcriptional Factors	Gene Significance	
		Catechin	Proanthocyanin
*ZjbZIP22841*	bZIP	0.860	0.865
*ZjERF7151*	ERF	0.859	0.887
*ZjERF9811*	ERF	0.856	0.834
*ZjHB-other16810*	HB-other	0.846	0.858
*ZjbZIP28585*	bZIP	0.807	0.855
*ZjNAC20984*	NAC	0.751	0.845
*ZjERF9866*	ERF	0.804	0.839
*ZjbZIP20176*	bZIP	0.800	0.828
*ZjERF31593*	ERF	0.793	0.790
*ZjWRKY17836*	WRKY	0.784	0.846
*ZjNAC20985*	NAC	0.751	0.845
*ZjMIKC19478*	MIKC	0.719	0.837
*ZjSBP10399*	SBP	0.782	0.814
*ZjbHLH15549*	bHLH	0.935	0.854
*ZjERF2360*	ERF	0.906	0.869
*ZjMYB18846*	MYB	0.867	0.857
*ZjC4H4518*	C4H	0.735	0.742
*ZjFLS11554*	FLS	0.548	0.602
*ZjUGT8470*	UGT	0.721	0.693
*ZjUGT4487*	UGT	0.691	0.760
*ZjUGT8359*	UGT	0.766	0.741
*ZjGST22051*	GST	0.777	0.664
*ZjGST22048*	GST	0.890	0.819
*ZjGST20311*	GST	0.821	0.870
*ZjGST0310*	GST	0.861	0.828

## Data Availability

No new data were created or analyzed in this study. Data sharing is not applicable to this article.
